# Prescribing patterns of long-acting injectable antipsychotics in a community setting in South Africa

**DOI:** 10.4102/sajpsychiatry.v28i0.1809

**Published:** 2022-06-30

**Authors:** Nabila Veyej, Mahomed Y.H. Moosa

**Affiliations:** 1 Department of Psychiatry, Faculty of Health Sciences, University of the Witwatersrand, Johannesburg, South Africa

**Keywords:** long-acting injectable antipsychotics, community clinics, nonadherence, co-prescribing, Africa

## Abstract

**Background:**

Long-acting injectable antipsychotics (LAI – APs) improve adherence to antipsychotics and decrease functional decline in schizophrenia. Yet they are prescribed late, in patients with established functional decline. Although LAI – APs are widely prescribed in South Africa, there is a paucity of research regarding the prescription profile for LAI – APs.

**Aim:**

This study aimed to describe prescribing practices for LAI – APs at psychiatric clinics.

**Setting:**

Community psychiatric clinics in South Africa.

**Methods:**

A retrospective review of the psychiatric files of all patients on LAI – APs attending the clinics over the study period was conducted. Sociodemographic, clinical and pharmacological information regarding the LAI – AP prescribed was extracted from the files.

**Results:**

A total of 206 charts were examined. The mean age of the study population was 46 (SD ± 12) years. Significantly more patients were male (*n* = 154; 74.8%), single (*n* = 184, 89.3%) and unemployed (*n* = 115; 55.8%) (*p* < 0.001). Approximately half had a comorbid substance use disorder (47.6%). The most common indication for the prescription of a LAI – AP was non-adherence (66%). Only 9.7% of the patients were prescribed a LAI – AP alone. No significant socio-demographic or clinical characteristic was associated with this prescribing habit. A LAI – AP was prescribed in combination with an oral antipsychotic, mood stabiliser or antidepressant in 53.9%, 44.7% and 7.8% of patients, respectively.

**Conclusion:**

Long-acting injectable antipsychotics were prescribed mainly following noncompliance with oral antipsychotics and may represent a missed opportunity to prevent functional decline. The high prevalence of LAI – AP polypharmacy has been highlighted.

## Introduction

The introduction of oral antipsychotic medication in the early 1950s transformed the treatment of schizophrenia globally.^1^ However, the problem of nonadherence or partial adherence to oral antipsychotic medications is common. Up to 50% of patients with schizophrenia do not take their oral medication as prescribed, resulting in a four-fold higher risk of relapse.^2,3^

Although most of the evidence regarding adherence to antipsychotic medication is derived from studies in patients with schizophrenia, the goal of treatment for any of the disorders in the schizophrenia spectrum and related disorders section of DSM 5^4^ is to prevent relapses, because relapses are associated with serious psychosocial, biological and economic consequences.^5^ After each relapse, it takes longer to achieve remission, there is a risk of treatment failure and there is evidence of progressive structural damage to the brain. Although the time taken to relapse after treatment discontinuation is variable, relapse is inevitable in the vast majority of patients after a period of approximately 2 years.^2,3,5,6^ Therefore, research to identify factors associated with nonadherence to antipsychotic medication is important.^3^

Several patient, provider and medication-related factors are associated with nonadherence or partial adherence to antipsychotic medication.^3^ One such medication-related factor, the prescribing of long-acting injectable formulations of antipsychotic medication, has been shown to improve adherence to antipsychotic medication.^3,7^ Long-acting injectable antipsychotics (LAI – APs) decrease the potential for missed doses because they are administered either once or twice per month, compared with the daily ingestion of an oral antipsychotic. Long-acting injectable antipsychotics also provide continuous drug delivery with a more favourable pharmacokinetic profile, higher bioavailability and stable receptor occupancy.^8^

Several studies have shown a definite advantage of LAI – APs in reducing relapses.^9,10,11^ However, some of the early studies in favour of LAI – APs over oral medications were called into question and some subsequently conducted randomised control trials (RCTs) failed to confirm a clear advantage of LAI – APs over oral antipsychotics.^12,13^ A possible explanation for these inconsistent findings is the methodological differences and bias associated with observational studies compared with RCTs. Compared with oral antipsychotics, the improved adherence associated with the prescription of LAI – APs has been shown to decrease the relapse rate^11^ and the number and duration of hospitalisations^14^ with a consequent decrease in the cost of care.^15^ Despite the improved adherence and the subsequent benefits conferred by LAI – APs, they are on average only prescribed in 20% – 37% of patients with schizophrenia.^7,16,17^

Various sociodemographic and clinical factors have been associated with the prescribing of LAI – APs. These factors include the duration of illness,^18^ gender,^18,19,20,21^ ethnicity,^22,23^ education,^23^ socio-economic status,^21,23,24,25^ comorbid substance use disorders,^23^ inpatient versus outpatient care,^20^ acceptability to patients,^26^ adherence to antipsychotic medication^3,7^ and attitude of psychiatrists towards LAI – APs.^27^

A key advantage of LAI – APs over oral antipsychotics is that they improve adherence to antipsychotic medication because they do not have to be taken daily. Yet, it is reported internationally that the concurrent prescription of oral psychotropic medication is frequent, with 46% – 75% of patients on LAI – APs also receiving concurrent prescriptions of an oral psychotropic.^23,26,28,29,30,31^

It is important to examine rational drug utilisation, because inappropriate prescription of medication contributes to poor health outcomes. Rational prescription of psychotropic medication is especially relevant in low-income and middle-income countries to prevent wastage of precious mental health resources.^32^

Despite over two decades of research on prescribing practices for LAI – APs globally,^24^ there remains a gap in the literature concerning the prescription patterns for LAI – APs in South Africa (SA). Only a few local studies have included information on the prescription of LAI – APs, and these were either limited to patients of only one ethnic group,^33^ patients recently discharged from a psychiatric hospital^34^ or outpatients attending tertiary psychiatric hospital clinics.^26^

Hence, the aim of this study was to examine the prescribing practices for LAI – APs in patients attending community psychiatric clinics in Johannesburg, SA. The general objective was to describe the sociodemographic characteristics, clinical characteristics, types, dosages and frequency of the LAI – APs that were prescribed. The specific objective was to determine the prevalence of concurrent prescriptions of LAI – APs and oral psychotropic medications and to determine if any significant sociodemographic and clinical characteristic were associated with these prescriptions.

## Method

This retrospective chart review was conducted at three psychiatric clinics based in the Chiawelo, Westbury and Lenasia South Community Health Centres. These clinics were randomly selected from the 23 clinics in the Johannesburg Metropolitan Health District. Three clinics were chosen because together they yielded a sufficient sample size. Each clinic in the district was assigned a number and three numbers were randomly drawn. The numbers drawn corresponded to the clinics included in this study. The psychiatric clinics are staffed by psychiatric nurses and medical officers or registrars in training for psychiatry. The psychiatric clinics’ patient records are filed separately from the community health center’s clinical records.

All adult patients (aged 18 and older) attending the clinics who had received a LAI – AP over a 1-month period were included in the study. There were no exclusion criteria.

Each clinic’s nursing staff maintains a monthly register of all patients for whom a LAI – AP is administered. The principal investigator accessed the clinical files of the patients who met the inclusion criteria and used a data collection form to manually record the following sociodemographic, clinical and treatment variables from the clinical notes: age, gender, population group, relationship status, the highest level of education achieved, employment status, primary psychiatric diagnosis, duration of illness, family history of mental illness, comorbid illnesses (psychiatric, medical and substance use), prescription of oral medication and details of the LAI – AP prescribed (the main indication for initiation, the date of initiation of the LAI – AP prescribed, the frequency of administration and the dose).

All records made on the data collection sheet were kept confidential. The name and patient numbers were anonymised on the populated databases in Microsoft Excel and raw data were only viewed by the principal investigator.

## Data analysis

A descriptive analysis of the sociodemographic and clinical variables was performed. All statistical analyses were conducted using R software (version 3.50, http://www.R-project.org). All tests were two-tailed probability values, and statistical significance was accepted when *p* ≤ 0.05. The data set for this study was generated from assessments of categorical scores. Categorical data are usually non-normal. Thus, nonparametric chi-squared contingency tests were used to analyse count data per variable.

For the purposes of meaningful statistical analysis, the patients were subsequently divided into two groups, namely those on LAI – AP alone and those on LAI – APs combined with oral antipsychotic medication, and the categories of some of the variables were collapsed. The chi-square test or Fisher’s exact test was applied to the categorical variables to determine if there was a significant relationship between the sociodemographic and clinical characteristics in patients receiving LAI – APs only versus those receiving concurrent prescriptions of LAI – APs and oral psychotropic medications. In the few cases of continuous data, these were tested for normality using QQ-plots and then analysed using *t*-tests for comparisons between the two groups. Multivariate logistic regression analysis was performed with 95% confidence intervals to adjust for relevant covariates.

### Ethical considerations

Ethical approval was obtained from the University of the Witwatersrand Human Research Ethics Committee (reference number: M181062). Permission to conduct the study was obtained from the research committee of Johannesburg Health District. All records made on the data collection sheet were kept confidential. The name and patient numbers were anonymised in the populated databases in Microsoft Excel and raw data were only viewed by the principal investigator.

## Results

This study comprised 206 patients who received a LAI – AP. Patients’ ages were grouped together by decade and ages ranged from 23 to 74 years, with a mean of 46 (SD ± 12) years. Significantly more patients were in the 51–60 year age group (*n* = 57; 27.7%), male (*n* = 154; 74.8%), black (*n* = 155; 75.2%), single (*n* = 184, 89.3%), had a secondary level of education (*n* = 106; 51.5%) and were unemployed (*n* = 115; 55.8%) (*p* < 0.001) (Table 1).

**TABLE 1:** Sociodemographic and clinical characteristics of the study population.

Variables	(*n* = 206)		*p*
	*n*	%	
**Gender**			*p* < 0.001
Male	154	74.8	
Female	52	25.2	
**Age (years)**			*p* < 0.001
Mean age 46 (SD ± 12) years			
18–30	24	11.7	
31–40	50	24.3	
41–50	43	20.9	
51–60	57	27.7	
≥ 60	32	15.5	
**RACE**			*p* < 0.001
Black	155	75.2	
Mixed race	31	15.0	
White	14	6.8	
Indian	6	2.9	
**Marital status**			*p* < 0.001
Single	184	89.3	
Married	9	4.4	
Divorced	12	5.8	
Widowed	1	0.5	
**Education**			*p* < 0.001
None	5	2.4	
Primary	60	29.1	
Secondary	106	51.5	
Tertiary	3	1.5	
Unknown	32	15.5	
**Employment status**			*p* < 0.001
Employed	8	3.9	
Unemployed	115	55.8	
Disability grant	55	26.7	
Old age pension	27	13.1	
Medically boarded	1	0.5	
**Primary psych disorder**			*p* < 0.001
Schizophrenia spectrum and related disorder	144	69.9	
Bipolar disorder	25	12.1	
Depressive disorder	4	1.9	
Intellectual disability	19	9.2	
Neurocognitive disorder	2	1.0	
Substance use disorder	12	5.8	
**Duration of illness (years)**			*p* < 0.001
Mean duration of illness19 (SD ± 11.5) years			
< 3	4	1.9	
3–5	20	9.7	
6–10	40	19.4	
11–20	55	26.7	
21–30	50	24.3	
> 30	35	17.0	
Unknown	2	1.0	
**Comorbid psych illness**	42	20.4	
**Comorbid substance use**	98	47.6	
**Comorbid medical illness**	68	33.0	

Significantly, more patients had a primary psychiatric diagnosis from the schizophrenia spectrum and related disorders chapter of the DSM 5 (*n* = 144; 69.9%) and a duration of illness of > 5 years (*n* = 180; 87.3%) (*p* < 0.001). A comorbid psychiatric illness and medical illness was present in 20.4% (*n* = 42) and 33% (*n* = 68) of the patients on a LAI – AP, respectively. The prevalence of comorbid substance abuse was 47.6% (*n* = 108) (Table 1).

The LAI – APs prescribed were either flupenthixol decanoate (*n* = 132; 64.1%) or zuclopenthixol decanoate (*n* = 74; 35.9%). The frequency of either form of injection was monthly (*n* = 197; 95.6%) or two-weekly (*n* = 9; 4.4%). The mean duration of prescription was 11.58 (SD ± 10.3) years, with the majority (*n* = 119; 57.8%) being on it for less than 19 years. The indications for prescribing an LAI – AP were nonadherence to oral medication (*n* = 136; 66%), followed by low level of functioning (*n* = 17; 8.3%), comorbid substance use (*n* = 16; 7.8%), nonresponse to orals (*n* = 12; 5.8%) and patient preference (*n* = 14; 6.8%). The dose of flupenthixol decanoate ranged from 10 mg to 80 mg, with a mean of 29.0 mg (SD ± 12.4). The most frequently prescribed monthly dose was 20 mg (*n* = 57; 43.2%). The dose of zuclopenthixol decanoate ranged from 80 mg to 800 mg with a mean of 271 mg (SD ± 107). The most frequently prescribed monthly dose was 200 mg (*n* = 32; 43.2%) (Table 2).

**TABLE 2:** Treatment characteristics of the study population.

Variables	(*n* = 206)		SD	*p*
	*N*	%		
**Type of LAI – AP prescribed**				*p* < 0.001
Flupenthixol	132	64.1	-	
Zuclopenthixol	74	35.9	-	
**Duration of prescription**				*p* = 0.69
Mean 11.58 years (SD ± 10.3)	-	-	-	
0–19 years	119	57.8	-	
20–29 years	50	24.3	-	
> 30 years	37	17.9	-	
**Frequency of dose**				*p* < 0.001
Monthly	197	95.6	-	
Two-weekly	9	4.4	-	
**Mean dose (mg)**				
Flupenthixol	29.0 mg	-	± 12.4	
Zuclopenthixol	271 mg	-	± 107	
**Indication for prescription**				*p* < 0.001
Nonadherence to oral psychotropics	136	66.0	-	
Low level of functioning	17	8.3	-	
Treatment resistance	17	8.3	-	
Intolerable side effects	1	0.5	-	
Comorbid substance use	16	7.8	-	
Patient preference	14	6.8	-	
Not recorded	5	2.4	-	

LAI – AP, long-acting injectable antipsychotics.

Of the 206 patients in the study population, only 9.7% (*n* = 20) were prescribed a LAI – AP alone. The other 90.3% (*n* = 186) was prescribed some oral psychotropic medication in addition to the LAI – AP. In more than half of these patients, it was an oral antipsychotic (*n* = 111, 53.9%). A mood stabiliser (*n* = 92; 44.7%) or antidepressant (*n* = 16; 7. 8%) was prescribed less frequently (Table 3).

**TABLE 3:** Concurrent prescription of long-acting injectable antipsychotics and psychotropic medication.

Variables	*n* = 206	
	*n*	%
LAI – AP only	20	9.7
LAI – AP combined with an oral antipsychotic	111	53.9
LAI –AP combined with a mood stabiliser	92	44.7
LAI – AP combined with an antidepressant	16	7.8
LAI – AP combined with any psychotropic medication	186	90.3

LAI – AP, long-acting injectable antipsychotics.

The sociodemographic and clinical characteristics of the patients on a LAI – AP only were compared with those on a LAI – AP combined with and an oral antipsychotic. There were no statistically significant differences between the group that was on LAI – AP alone compared with the group that was on LAI – AP combined with an oral antipsychotic with respect to gender (*x*^2^ = 0.435; *p* = 0.5), age (*x*^2^ = 3.845; *p* = 0.1), the mean age (*t* = −1.12, *p* = 0.3), race (*x*^2^ = 1.665; *p* = 0.2), marital status (*p* = 0.359), level of education achieved (*x*^2^ = 1.526; *p* = 0.5), employment status (*p* = 0.59), primary psychiatric diagnosis (*p* = 0.363), duration of illness (*p* = 0.346), the mean duration of illness (*t* = 0.76, *p* = 0.5) and the presence of comorbid psychiatric illness (*x*^2^ = 1.813; *p* = 0.2) or comorbid substance use (*x*^2^ = 1.018; *p* = 0.3) (Table 4). There was a statistically significant difference with regard to comorbid medical illness (*x*^2^ = 4.979, *p* = 0.03) (Table 4). However, on multivariate analysis, the *p*-value was 0.6.

**TABLE 4:** Frequency distribution of the sociodemographic and clinical characteristics of patients on long-acting injectable antipsychotics (LAI – APs) only, compared with those on LAI – AP combined with oral antipsychotics.

Variables	LAI – AP only (*n* = 20)			LAI – AP and oral antipsychotic (*n* = 111)			Statistics
	*N*	%	SD	*N*	%	SD	
**Gender**							
Male	16	80.0	-	81	72.9	-	Χ^2^ = 0.435 *p* = 0.5
Female	4	20.0	-	30	27.0	-	
**Age (years)**							
Mean age (years)	42	-	± 11	45	-	± 13	*t* = −1.12, *p* = 0.3
18–39	9	45.0	-	45	40.5	-	Χ^2^ = 3.845 *p* = 0.1
40–59	11	55.0	-	48	43.2	-	
> 60	0	0.0	-	18	16.2	-	
**Race**							
Black	17	85.0	-	79	71.2	-	Χ^2^ = 1.655 *p* = 0.2
Nonblack	3	15.0	-	32	28.8	-	
**Marital status**							
Married	0	0.0	-	10	9.0	-	0.359*
Single	20	100	-	101	90.9	-	
**Highest level of education**							
None or primary	4	20.0	-	35	31.5	-	Χ^2^ = 1.526 *p* = 0.5
Secondary or tertiary	11	55.0	-	58	52.3	-	
Unknown	5	25.0	-	18	16.2	-	
**Employment status**							
Employed	0	0.0	-	6	5.4	-	*p* = 0.590*
Unemployed or on a grant	20	100	-	105	94.6	-	
**Primary psych disorder**							
Schizophrenia spectrum	18	90.0	-	75	67.6	-	*p* = 0.363*
Mood disorder	1	5.0	-	12	10.8	-	
Intellectual disability/neurocognitive	1	5.0	-	16	14.4	-	
Substance use disorder	0	0.0	-	8	7.2	-	
**Duration of illness**							
Mean duration of illness (years)	17	-	± 12	19	-	± 12	*t* = 0.76, *p* = 0.5
0–19 years	13	65.0	-	87	78.4	-	*p* = 0.346*
20–29 years	5	25.0	-	15	13.5	-	
> 30 years	1	5.0	-	8	7.2	-	
Unknown	1	5.0	-	1	0.9	-	
Comorbid psych illness	13	65.0	-	54	48.6	-	Χ^2^ = 1.813 *p* = 0.2
Comorbid substance use	12	60.0	-	53	47.7	-	Χ^2^ = 1.018 *p* = 0.3
Comorbid medical illness	2	10.0	-	39	35.1	-	Χ^2^ = 4.979 *p* = 0.03

LAI – AP, long-acting injectable antipsychotics.

* , Fisher’s exact test.

The treatment characteristics of the patients on a LAI – AP only were compared with those on a LAI – AP combined with an oral antipsychotic. There was also no statistically significant difference between the two groups with regard to the specific LAI – AP prescribed (*x*^2^ = 0.027; *p* = 0.87), the mean duration of the LAI – AP prescribed (*t* = 1.8; *p* = 0.07), the mean dose of flupenthixol (*t* = 0.96; *p* = 0.3), the mean dose of zuclopenthixol (*t* = 0.49; *p* = 0.6), the injection interval (*p* = 1.000) and the indications for LAI – AP prescription (*p* = 0.912) (Table 5).

**TABLE 5:** Frequency distribution of the treatment characteristics in the group of patients on long-acting injectable antipsychotics (LAI – APs) only compared with the group on LAI – APs combined with oral antipsychotics.

Variables	LAI – AP only (*n* = 20)				LAI – AP and oral antipsychotic (*n* = 111)				Statistics
	*N*	%	Mean	*SD*	*N*	%	Mean	*SD*	
**Type prescribed**									
Flupenthixol	13	65	-	-	70	63.1	-	-	Χ^2^ = 0.027 *p* = 0.87
Zuclopenthixol	7	35.0	-	-	41	36.9	-	-	
**Duration of prescription (years)**			8	± 6			13	± 11	*t* = 1.8, *p* = 0.07
0–19 years	13	65.0	-	-	87	78.4	-	-	*p* = 0.346*
20–29 years	5	25.0	-	-	15	13.5	-	-	
> 30 years	2	10.0	-	-	9	8.1	-	-	
**Frequency of dose**									
Monthly	20	100	-	-	106	95.5	-	-	*p* = 1.000*
Two-weekly	0	0	-	-	5	4.5	-	-	
**Mean dose (mg)**									
Flupenthixol	32.7	-	-	± 14.2	28.71	-	-	± 12.7	*t* = −0.96, *p* = 0.3
Zuclopenthixol	244.4	-	-	± 101.3	263.9	-	-	± 107.2	*t* = −0.49, *p* = 0.6
**Indication for prescription**									
Nonadherence	15	75	-	-	69	62.2	-	-	*p* = 0.912*
Low level of functioning	1	5.0	-	-	9	8.1	-	-	
Treatment resistance	0	0.0	-	-	4	3.6	-	-	
Intolerable side effects	0	0.0	-	-	1	0.9	-	-	
Comorbid substance use	2	10.0	-	-	10	9.0	-	-	
Long untreated psychosis	0	0.0	-	-	0	0.0	-	-	
Nonresponse to orals	0	0.0	-	-	8	7.2	-	-	
Patient preference	1	5.0	-	-	7	6.3	-	-	
Not recorded	1	5.0	-	-	3	2.7	-	-	

LAI – AP, long-acting injectable antipsychotics.

* , Fisher’s exact test.

## Discussion

The prescription pattern for LAI – APs in this study is in keeping with the traditional profile of patients for whom LAI – APs are prescribed globally.^25,35^ Long-acting injectable antipsychotics have long been prescribed for patients who are older, male, not compliant with oral medication, inclined to relapse frequently and potentially posing a danger to others.^17^ In this study, LAI – APs were prescribed mostly for patients who were over the age of 40 (64.1%), male (74.8%), single (89.3%), unemployed or in receipt of a social grant (96.1%), diagnosed with a condition from the schizophrenia and related disorders chapter of DSM 5( 69.9%) and experiencing a duration of illness greater than 5 years (88.4%). Flupenthixol decanoate was the most frequently prescribed LAI – AP (64.1%), and the main indication for a LAI – AP was nonadherence to oral antipsychotic medication (66%). Polypharmacy was common; 90% of the patients were on a LAI – AP combined with an oral psychotropic.

The mean age of the patients in this study was 46 years. This is in keeping with a systematic review that reported a mean age at treatment with LAI – APs ranging from 38.5 years in the 1970s to 39.5 years in the 2000s,^25^ a study conducted across six East Asian countries,^18^ and similar to three publications from Italy,^35^ the United States of America (USA)^36^ and Australia.^37^

Three-quarters of the patients on LAI – APs in this study were males. Although gender disparity in the prescription of LAI – APs is a consistent finding in the literature,^25^ the proportion of males receiving a LAI – AP was higher than that reported by Lindstrom et al. (58%)^19^ and Sim et al. (56%).^18^ A possible explanation may be the high frequency (66%) of nonadherence as the indication for initiation of the LAI – AP in this study. Women generally have higher adherence rates to psychotropic medications than men.^38^ It is also possible that prescribers utilise coercive treatment for male patients more readily.

Only 1.5% of patients who were prescribed a LAI – AP in this study had a tertiary level of education and 3.9% were employed. This is in stark contrast to a study from Europe, where 44% of patients on a LAI – AP had a university degree and 22% were employed.^35^ However, this is most likely a reflection of the high rate of unemployment amongst patients with mental illness in SA^34^ and because most of these higher functioning patients were receiving second-generation antipsychotic (SGA) LAI – APs.

The diagnostic indication for a LAI – AP in this study was consistent with several studies that reported that LAI – APs are most frequently prescribed for patients with schizophrenia.^9,18,27,35,36^ Schizophrenia is a chronic mental illness that is associated with poor adherence to oral medication resulting in a high risk of relapse and admission to hospital.^3,38^ Adherence to antipsychotic medication is critical for the recovery and restoration of occupational and social dysfunction in schizophrenia.^3^ Several studies have confirmed that prescribing LAI – APs is an effective strategy to improve treatment adherence in schizophrenia.^10,24^ Consequently, several international schizophrenia management guidelines^10,17,39^ and the Standard Treatment Guidelines and Essential Medicines List for SA^40^ recommend the prescription of LAI – APs in schizophrenia. Hence, the prescribing of LAI – APs was therefore rational and in accordance with these guideline recommendations and the manufacturer’s labelled indication for LAI – APs.^41^

Approximately 10% of the patients who were prescribed LAI – APs in this study had a primary diagnosis of bipolar disorder. This figure is similar to the range of 4.8% – 8.0% reported in other studies^22,30,37^ but lower than the 18% reported by Ostuzzi et al.^35^ Bipolar disorder is a potentially lifelong chronic mental illness and adherence to medication is also critical for effective control of symptoms, prevention of relapse and rehospitalisation.^42,43^ Recently, the indications for LAI – APs have been expanded beyond schizophrenia to include approval for use in the maintenance phase of bipolar disorder. It has been shown that LAI – APs are also effective in reducing relapse rates in patients with bipolar disorder who are poorly adherent to medication.^43^ Consequently, the prescription of LAI – APs as both monotherapy and adjunctive therapy to lithium or valproate for the maintenance treatment of bipolar disorder has been approved by some guidelines, including that of the Canadian Network for Mood and Anxiety Treatments,^44^ the United States Food and Drug Administration and the European Medical Agency.^43^

However, it is only the SGA LAI – APs, and not the first-generation (FGA) LAI – APs, that are approved for use in bipolar disorder.^42^ Yet, in this study, FGA LAI – APs were prescribed for bipolar disorder. As previously discussed, because of the lack of availability of SGA LAI – APs on The South African National Department of Health Standard Treatment Guidelines and Essential Medicines List, it is likely that first-generation LAI – APs became the only available pharmacological option for patients with bipolar disorder who were not adherent to oral medication.

Only 12% of the patients on a LAI – AP in this study had a duration of illness that was less than 5 years. The first 5 years after the onset of the illness has been called the ‘critical period’ because most of the clinical and psychosocial decline occurs within this period.^45^ There is little doubt that nonadherence to treatment is a predictor of symptom recurrence in patients with a first episode of psychosis.^6^ Several authors have therefore suggested that LAI – APs should be prescribed in patients with a first episode of psychosis, because the prescription of LAI – APs in younger patients after a first episode of schizophrenia has been shown to improve adherence, prevent relapses, reduce hospitalisations and result in better overall long-term outcomes.^7,10,17,46,47^ It is possible that doctors’ reluctance to prescribe LAI – APs in patients with a first episode psychosis in this study may have been influenced by their attitudes towards LAI – APs. A study from the United Kingdom found that individual psychiatrists were reluctant to prescribe LAI – APs because they viewed LAI – APs as coercive, stigmatising and less acceptable to patients.^27^ Even in Africa, where medical paternalism is common, some psychiatrists still viewed LAI – APs as coercive.^48^

A comorbid substance use disorder was present in almost half of the patients (48%) on LAI – APs in this study. There is little doubt that individuals with comorbid substance use have a consistently higher prevalence of nonadherence to antipsychotic medication than those without.^3,38^ Although the evidence is not robust, there is proof of improved adherence and symptom control in patients on LAI – APs with dual diagnoses,^46^ with better outcomes for SGA LAI – APs compared with FGA LAI –APs.^49^

Flupenthixol decanoate was prescribed more frequently than zuclopenthixol decanoate in this study (64% vs. 36%). This preference for flupenthixol over zuclopenthixol was also reported in New Zealand,^50^ Australia^51^ and Nigeria.^31^ Although earlier studies reported that zuclopenthixol decanoate had greater antiaggressive efficacy and flupenthixol decanoate had greater antidepressant properties,^24^ subsequent systematic reviews found no real differences in global outcome measures or adverse events between them.^52^ The choice of prescription between the two LAI – APs in this study was therefore most probably because of a local prescribing habit, general availability and familiarity with the drug.^36^ In the public sector of SA, only FGA LAI – APs are available on the national formulary.^40^ Second-generation antipsychotic LAI – APs are obtainable on a named patient basis, by prior motivation through a buy-out process. However, a recent systematic review and meta-analysis found no difference in efficacy and discontinuation rates between first-generation compared with second-generation LAI – APs in the management of schizophrenia.^53^

The main indication for using LAI – APs was nonadherence to oral psychotropic medication. Yet, this study found that only 9.7% of the patients were prescribed a LAI – AP alone. The remaining 90.3% were prescribed an oral psychotropic medication (another antipsychotic and a mood stabiliser and an antidepressant) in combination with the LA – AP.

In approximately half of the patients, the other oral psychotropic medication coprescribed was an antipsychotic. Similar high prescription rates have been reported in clinical practice globally, including several studies from the United States of America,^23,28,29,30,54^ Nigeria,^31^ SA^26^ and Korea.^55^ The authors explain that some of the patients in these studies had recently been discharged from psychiatric institutions and that either the oral antipsychotic supplementation was used to augment the LAI – AP during the period of stabilisation or that they were being ‘weaned off’ oral antipsychotics that had been initiated during hospitalisation.^23,30,54^ However, our finding is significant because ours was a community clinic-based sample of patients, many of whom had been prescribed this combination for prolonged periods.

Antipsychotic polypharmacy (combining two antipsychotic medications, i.e. LAI – APs with oral antipsychotic medication) is a questionable prescribing habit because it argues against the main indication of nonadherence and increases the potential for drug–drug interactions, a range of side effects and cost of care.^16^ However, because the practice is so widespread in real-world settings, it is possible that the response to LAI – APs monotherapy in published clinical trials is overestimated. It may also be a reflection of the illness severity and complexity in patients for whom LAI – APs are prescribed.^28^ Our study did not find any significant sociodemographic and clinical characteristics associated with this prescribing habit.

Similar to the high rate of concurrent oral antipsychotic prescriptions, approximately half of the patients in this study (44.7%) were prescribed a mood stabiliser in addition to the LAI – AP. Our figure is in keeping with those in previous reports of concurrent mood stabiliser coprescriptions ranging from 19%^28^ to 47%.^54^ It is possible that in our study, the mood stabiliser was used to augment the LAI – AP in patients with treatment resistance who had a poor response to antipsychotic medication^56^ or that LAI – APs were used in nonadherent patients with bipolar disorder because an injectable mood stabiliser is not yet available or for the treatment of the bipolar phase of schizoaffective disorder.

About 8% of the study population was prescribed an antidepressant in addition to the LAI – AP. Although Joo et al.^55^ and Olfson et al.^54^ also reported similar prescribing patterns, the findings cannot be directly compared with our study because concurrent antidepressant use in their studies was in the first few months after initiation of a LAI – AP only.

This study was unable to establish any significant sociodemographic and clinical characteristics that were associated with the prescribing of LAI – AP combined with oral psychotropic medication. There is a paucity of published research regarding this comparison with Doshi et al.^29^ also having reported no association. However, Aggarwal et al.^28^ reported that patients taking concomitant LAI – APs and oral psychotropic medications were more likely to abuse alcohol. The authors could not account for this finding, except to note that prescribers may have thought that patients with alcohol abuse were more likely to be unreliable and have cognitive impairment.

## Limitations

Limitations of our study include the lack of generalisability to other community clinics in SA, incomplete clinical records and the cross-sectional study design. The lack of information regarding the prescription of LAI – APs for specific diagnoses such as schizoaffective disorder and symptom severity is a further limitation, because this could have been a possible explanation for the high prevalence of polypharmacy.

To the best of our knowledge, this is the first report of prescription patterns for LAI – APs in a community health setting in SA. Globally, there is minimal information regarding prescription patterns of LAI – APs in community health settings.^36^ Therefore, the strengths of the study are the community clinic setting and the information on real-world patients in usual care, given the general lack of data on antipsychotic utilisation in clinical practice from Africa.^57^ Furthermore, this study provides some information on the prevalence of combining LAI –APs and oral psychotropic treatment, which is not well established in low-income and middle-income countries with restricted formularies.

## Conclusions

The results of this study confirm that despite calls for the increased utilisation of LAI – APs in the earlier phases of schizophrenia,^7,10,17,46^ LAI – APs in SA were prescribed mainly for older male patients who were not gainfully employed, with dual diagnoses and who were not adherent to oral medication. The authors highlight the high prevalence of polypharmacy in patients receiving LAI – APs and the lack of SGA LAI – APs in community psychiatric clinics in Johannesburg. Further research is needed to establish the prescribing habits for LAI – APs in other provinces in SA and to investigate the adherence and quality of life of patients on LAI – APs compared with oral antipsychotics.

## References

[1] Cunningham Owens D, Johnstone EC. The development of antipsychotic drugs. Brain Neurosci Adv. 2018;2:239821281881749. 10.1177/2398212818817498 PMC7058266 32166169

[2] Alvarez-Jimenez M, Priede A, Hetrick SE, et al. Risk factors for relapse following treatment for first episode psychosis: A systematic review and meta-analysis of longitudinal studies. Schizophr Res 2012;139(1–3):116–128. 10.1016/j.schres.2012.05.007 22658527

[3] Kane JM, Kishimoto T, Correll CU. Non-adherence to medication in patients with psychotic disorders: Epidemiology, contributing factors and management strategies. World Psychiatry. 2013;12(3):216–226. 10.1002/wps.20060 24096780 PMC3799245

[4] American Psychiatric Association. Diagnostic and statistical manual of mental disorders. DSM–5. 5th ed. Arlington, TX: American Psychiatric Association; 2013.

[5] Emsley R, Chiliza B, Asmal L, Harvey BH. The nature of relapse in schizophrenia. BMC Psychiatry. 2013;13:1–8. 10.1186/1471-244X-13-50 23394123 PMC3599855

[6] Zipursky RB, Menezes NM, Streiner DL. Risk of symptom recurrence with medication discontinuation in first-episode psychosis: A systematic review. Schizophr Res. 2014;152(2–3):408–414. 10.1016/j.schres.2013.08.001 23972821

[7] Emsley R, Chiliza B, Asmal L, Mashile M, Fusar-Poli P. Long-acting injectable antipsychotics in early psychosis: A literature review. Early Interv Psychiatry. 2013;7(3):247–254. 10.1111/eip.12027 23342964

[8] Ereshefsky L, Mascarenas CA. Comparison of the effects of different routes of antipsychotic administration on pharmacokinetics and pharmacodynamics. J Clin Psychiatry. 2003;64(Supp 16):18–23. 14680415

[9] Tiihonen J, Mittendorfer-Rutz E, Majak M, et al. Real-world effectiveness of antipsychotic treatments in a nationwide cohort of 29 823 patients with schizophrenia. JAMA Psychiatry. 2017;7(74):686–693. 10.1001/jamapsychiatry.2017.1322 PMC5710250 28593216

[10] Correll CU, Citrome LL, Haddad PM, et al. The use of long-acting injectable antipsychotics in schizophrenia: Evaluating the evidence. J Clin Psychiatry. 2016;77(suppl. 3):1–24. 10.4088/JCP.15032su1 27732772

[11] Leucht C, Heres S, Kane JM, Kissling W, Davis JM, Leucht S. Oral versus depot antipsychotic drugs for schizophrenia – A critical systematic review and meta-analysis of randomised long-term trials. Schizophr Res. 2017;127(1–3):83–92. 10.1016/j.schres.2010.11.020 21257294

[12] Kishimoto T, Robenzadeh A, Leucht C, et al. Long-acting injectable vs oral antipsychotics for relapse prevention in schizophrenia: A meta-analysis of randomized trials. Schizophr Bull. 2012;40(1):192–213. 10.1093/schbul/sbs150 23256986 PMC3885289

[13] Ostuzzi G, Bighelli I, So R, Furukawa TA, Barbui C. Does formulation matter? A systematic review and meta-analysis of oral versus long-acting antipsychotic studies. Schizophr Res. 2017;183:10–21. 10.1016/j.schres.2016.11.010 27866695

[14] Taipale H, Mehtälä J, Tanskanen A, Tiihonen J. Comparative effectiveness of antipsychotic drugs for rehospitalization in schizophrenia – A nationwide study with 20-year follow-up. Schizophr Bull. 2018;44(16):1381–1387. 10.1093/schbul/sbx176 29272458 PMC6192491

[15] Achilla E, McCrone P. The cost effectiveness of long-acting/extended-release antipsychotics for the treatment of schizophrenia: A systematic review of economic evaluations. Appl Heal Econ Heal Policy. 2013;11(2):95–106. 10.1007/s40258-013-0016-2 23494934

[16] Barnes TRE, Shingleton-smith A, Paton C. Antipsychotic long-acting injections: Prescribing practice in the UK. Br J Psychiatry. 2009;195(S52):s37–s42. 10.1192/bjp.195.52.s37 19880915

[17] Llorca PM, Abbar M, Courtet P, Guillaume S, Lancrenon S, Samalin L. Guidelines for the use and management of long-acting injectable antipsychotics in serious mental illness. BMC Psychiatry. 2013;13:1–17. 10.1186/1471-244X-13-340 24359031 PMC3898013

[18] Sim K, Su A, Ungvari GS, Fujii S, et al. Depot antipsychotic use in schizophrenia: An East Asian perspective. Hum Psychopharmacol. 2004;19(2):103–109. 10.1002/hup.571 14994320

[19] Lindström E, Wilderlöv B, Von Knorring L. Antipsychotic drugs-a study of the prescription pattern in a total sample of patients with a schizophrenic syndrome in one catchment area in the county of Uppland, Sweden, in 1991. Int Clin Psychopharmacol. 1996;11(4):241–246. 10.1097/00004850-199612000-00005 9031990

[20] Remington G, Shammi CM, Sethna R, Lawrence R. Antipsychotic dosing patterns for schizophrenia in three treatment settings. Psychiatr Serv. 2001;52(1):96–98. 10.1176/appi.ps.52.1.96 11141536

[21] Verdoux H, Pambrun E, Tournier M, Bezin J, Pariente A. Antipsychotic long-acting injections: A community-based study from 2007 to 2014 of prescribing trends and characteristics associated with initiation. Schizophr Res. 2016;178(1–3):58–63. 10.1016/j.schres.2016.09.014 27624680

[22] Aggarwal NK, Rosenheck RA, Woods SW, Sernyak MJ. Race and long-acting antipsychotic prescription at a community mental health center: A retrospective chart review. J Clin Psychiatry. 2012;73(4):513–517. 10.4088/JCP.11m07161 22579151 PMC3885178

[23] Shi L, Ascher-Svanum H, Zhu B, Faries D, Montgomery W, Marder SR. Characteristics and use patterns of patients taking first-generation depot antipsychotics or oral antipsychotics for schizophrenia. Psychiatr Serv. 2007;58(4):482–488. 10.1176/ps.2007.58.4.482 17412849

[24] Sreeraj VS, Shivakumar V, Rao NP, Venkatasubramanian G. A critical appraisal of long acting injectable antipsychotics translating research to clinics. Asian J Psychiatr. 2017;28(2017):57–64. 10.1016/j.ajp.2017.03.018 28784398

[25] Rossi G, Frediani S, Rossi R, Rossi A. Long-acting antipsychotic drugs for the treatment of schizophrenia: Use in daily practice from naturalistic observations. BMC Psychiatry. 2012;12(1):1. 10.1186/1471-244X-12-122 22909285 PMC3573926

[26] Roopun K, Tomita A, Paruk S. Attitude and preferences towards oral and long-acting injectable antipsychotics in patients with psychosis in KwaZulu-Natal, South Africa. S Afr J Psychiatr 2020;26:1–9. 10.4102/sajpsychiatry.v26i0.1509 PMC7433262 32832130

[27] Patel MX, David AS. Why aren’t depot antipsychotics prescribed more often? Adv Psychiatr Treat. 2005;11(3):203–211. 10.1192/apt.11.3.203

[28] Aggarwal NK, Sernyak MJ, Rosenheck RA. Prevalence of concomitant oral antipsychotic drug use among patients treated with long-acting, intramuscular, antipsychotic medications. J Clin Psychopharmacol. 2012;32(3):323–328. 10.1097/JCP.0b013e31825244f6 22544006

[29] Doshi JA, Pettit AR, Stoddard JJ, Zummo J, Marcus SC. Concurrent oral antipsychotic drug use among schizophrenia patients initiated on long-acting injectable antipsychotics post-hospital discharge. J Clin Psychopharmacol. 2015;35(4):442–446. 10.1097/JCP.0000000000000353 26075492

[30] Alastanos JN, Paxos C, Emshoff J. Evaluation of oral antipsychotic supplementation of select second-generation long-acting injectable antipsychotics in an acute-care psychiatric setting. Ment Health Clin. 2019;9(1):18–23. 10.9740/mhc.2019.01.018 30627499 PMC6322819

[31] Igbinomwanhia NG, Olotu SO, James BO. Prevalence and correlates of antipsychotic polypharmacy among outpatients with schizophrenia attending a tertiary psychiatric facility in Nigeria. Ther Adv Psychopharmacol. 2017;7(1):3–10. 10.1177/2045125316672134 28101318 PMC5228713

[32] Agbonile IO, Famuyiwa O. Psychotropic drug prescribing in a Nigerian psychiatric hospital. Int Psychiatry. 2009;6(4):96–98. 10.1192/S1749367600000795 31508010 PMC6734891

[33] Koen L, Magni P, Niehaus DJ, le Roux A. Antipsychotic prescription patterns in Xhosa patients with schizophrenia or schizoaffective disorder. Afr J Psychiatry. 2008;11:287–290. 19588052

[34] Armstrong KS, Temmingh H. Prevalence of and factors associated with antipsychotic polypharmacy in patients with serious mental illness: Findings from a cross-sectional study in an upper-middle-income country. Braz J Psychiatry. 2017;39(4):293–301. 10.1590/1516-4446-2016-2015 28177063 PMC7111406

[35] Ostuzzi G, Mazzi MA, Terlizzi S, et al. Factors associated with first- versus second-generation long-acting antipsychotics prescribed under ordinary clinical practice in Italy. PLoS One. 2018;13(8):1–17. 10.1371/journal.pone.0201371 PMC6072022 30071042

[36] Netley J, Gaul J, Ferguson C. Evaluation of prescribing patterns of long-acting injectable antipsychotics within a community health system. J Clin Psychopharmacol. 2019;39(5):494–498. 10.1097/JCP.0000000000001095 31425464

[37] Herriot P, Kheirani K. Survey of depot antipsychotic prescribing in southern Adelaide. Australas Psychiatry. 2003;13(3):253–258. 10.1080/j.1440-1665.2005.02196.x 16174198

[38] Lacro J, Dunn L, Dolder C, Leckband S, Jeste DV. Prevalence of and risk factors for medication nonadherence in patients with schizophrenia: A comprehensive review of recent literature. J Clin Psychiatry. 2002;63(10):892–909. 10.4088/JCP.v63n1007 12416599

[39] Canadian Psychiatric Association. Clinical practice guidelines: Treatment of schizophrenia. Can J Psychiatry. 2005;50:7–57.

[40] The South African National Department of Health. Standard treatment guidelines and essential medicines list for South Africa [homepage on the Internet]. 2020; p. 388–389. Available from: http://www.health.gov.za/edp.php

[41] Lundbeck. Product monograph flupentixol decanoate intramuscular injection; 2017. St Laurent, QC: Lundbeck Canada Inc; p. 1–35.

[42] Gigante AD, Lafer B, Yatham LN. Long-acting injectable antipsychotics for the maintenance treatment of bipolar disorder. CNS Drugs. 2012;26(5):403–420. 10.2165/11631310-000000000-00000 22494448

[43] Pacchiarotti I, Tiihonen J, Kotzalidis GD, et al. Long-acting injectable antipsychotics (LAIs) for maintenance treatment of bipolar and schizoaffective disorders: A systematic review. Eur Neuropsychopharmacol. 2019;29(4):457–470. 10.1016/j.euroneuro.2019.02.003 30770235

[44] Yatham L, Kennedy S, Parikh S, et al. Canadian Network for Mood and Anxiety Treatments (CANMAT) and International Society for Bipolar Disorders (ISBD) collaborative update of CANMAT guidelines for the management of patients with bipolar disorder: Update 2013. Bipolar Disord. 2013;15(1):1–44. 10.1111/bdi.12025 23237061

[45] Birchwood M, Todd P, Jackson C. Early intervention in psychosis: The critical period hypothesis. Br J Psychiatry. 1998;172(S33):53–59. 10.1192/S0007125000297663 9764127

[46] Abdel-Baki A, Thibault D, Medrano S, et al. Long-acting antipsychotic medication as first-line treatment of first-episode psychosis with comorbid substance use disorder. Early Interv Psychiatry. 2020;14(1):69–79. 10.1111/eip.12826 31125513

[47] Medrano S, Abdel-Baki A, Stip E, Potvin S. Three-year naturalistic study on early use of long-acting injectable antipsychotics in first episode psychosis. Psychopharmacol Bull. 2018;48(4):25–61. 30618474 10.64719/pb.4577 PMC6294417

[48] James BO, Omoaregba JO, Okonoda KM, Otefe EU, Patel MX. The knowledge and attitudes of psychiatrists towards antipsychotic long-acting injections in Nigeria. Ther Adv Psychopharmacol. 2012;2(5):169–177. 10.1177/2045125312453158 23983972 PMC3736947

[49] Rubio G, Martínez I, Ponce G, Jiménez-Arriero MA, López-Muñoz F, Álamo C. Long-acting injectable risperidone compared with zuclopenthixol in the treatment of schizophrenia with substance abuse comorbidity. Can J Psychiatry. 2006;51(8):531–539. 10.1177/070674370605100808 16933590

[50] Humberstone V, Wheeler A, Lambert T. An audit of outpatient antipsychotic usage in the three health sectors of Auckland, New Zealand. Aust N Z J Psychiatry. 2004;38(4):240–245. 10.1080/j.1440-1614.2004.01340.x 15038803

[51] Keks N, Altson K, Hope J, et al. Use of antipsychosis and adjunctive medications by an inner urban community psychiatric service. Aust N Z J Psychiatry. 1999;33(6):896–901. 10.1046/j.1440-1614.1999.00639.x 10619218

[52] Adams CE, Fenton MKP, Quraishi S, David AS. Systematic meta-review of depot antipsychotic drugs for people with schizophrenia. Br J Psychiatry. 2001;179:290–299. 10.1192/bjp.179.4.290 11581108

[53] Saucedo Uribe E, Carranza Navarro F, Guerrero Medrano AF, et al. Preliminary efficacy and tolerability profiles of first versus second-generation long-acting injectable antipsychotics in schizophrenia: A systematic review and meta-analysis. J Psychiatr Res. 2020;129(March):222–233. 10.1016/j.jpsychires.2020.06.013 32805530

[54] Olfson M, Marcus SC, Ascher-Svanum H. Treatment of schizophrenia with long-acting fluphenazine, haloperidol, or risperidone. Schizophr Bull. 2007;33(6):1379–1387. 10.1093/schbul/sbm033 17470444 PMC2779880

[55] Joo SW, Shon SH, Choi GJ, Koh MJ, Cho SW, Lee J. Continuation of schizophrenia treatment with three long-acting injectable antipsychotics in South Korea: A nationwide population-based study. Eur Neuropsychopharmacol. 2019;29(9):1051–1060. 10.1016/j.euroneuro.2019.07.138 31362852

[56] Sommer IE, Begemann MJH, Temmerman A, Leucht S. Pharmacological augmentation strategies for schizophrenia patients with insufficient response to clozapine: A quantitative literature review. Schizophr Bull. 2012;38(5):1003–1011. 10.1093/schbul/sbr004 21422107 PMC3446238

[57] Purgato M, Adams C, Barbui C. Schizophrenia trials conducted in African countries: A drop of evidence in the ocean of morbidity? Int J Ment Health Syst. 2012;6:9. 10.1186/1752-4458-6-9 22768830 PMC3447718

